# X-linked immunodeficient (XID) mice exhibit high susceptibility to *Cryptococcus gattii* infection

**DOI:** 10.1038/s41598-021-97041-9

**Published:** 2021-09-15

**Authors:** Israel Diniz-Lima, Pablo Rodrigo da Rosa, Elias Barbosa da Silva-Junior, Joyce Cristina Guimarães-de-Oliveira, Elisangela Oliveira de Freitas, Danielle de Oliveira Nascimento, Alexandre Morrot, Leonardo Nimrichter, Jose Osvaldo Previato, Lucia Mendonça-Previato, Leonardo Freire-de-Lima, Debora Decote-Ricardo, Celio Geraldo Freire-de-Lima

**Affiliations:** 1 Instituto de Biofísica Carlos Chagas Filho, Universidade Federal do Rio de Janeiro, Rio de Janeiro, 21941-900 Brazil; 2 Instituto de Veterinária, Universidade Federal Rural do Rio de Janeiro, Seropédica, 23890-000 Brazil; 3 Instituto Oswaldo Cruz, FIOCRUZ, Rio de Janeiro, 21045-900 Brazil; 4 Faculdade de Medicina, Universidade Federal do Rio de Janeiro, Rio de Janeiro, 21941-900 Brazil; 5 Instituto de Microbiologia Paulo de Góes, Universidade Federal do Rio de Janeiro, Rio de Janeiro, 21941-900 Brazil

**Keywords:** Fungal host response, Fungal pathogenesis

## Abstract

Cryptococcosis is an opportunistic disease caused by the fungus *Cryptococcus neoformans* and *Cryptococcus gattii*. It starts as a pulmonary infection that can spread to other organs, such as the brain, leading to the most serious occurrence of the disease, meningoencephalitis. The humoral response has already been described in limiting the progression of cryptococcosis where the B-1 cell seems to be responsible for producing natural IgM antibodies, crucial for combating fungal infections. The role of the B-1 cell in *C. neoformans* infection has been initially described, however the role of the humoral response of B-1 cells has not yet been evaluated during *C. gattii* infections. In the present study we tried to unravel this issue using XID mice, a murine model deficient in the Btk protein which compromises the development of B-1 lymphocytes. We use the XID mice compared to BALB/c mice that are sufficient for the B-1 population during *C. gattii* infection. Our model of chronic lung infection revealed that XID mice, unlike the sufficient group of B-1, had early mortality with significant weight loss, in addition to reduced levels of IgM and IgG specific to GXM isolated from the capsule of *C. neoformans*. In addition to this, we observed an increased fungal load in the blood and in the brain. We described an increase in the capsular size of *C. gattii* and the predominant presence of cytokines with a Th2 profile was also observed in these animals. Thus, the present study strongly points to a higher susceptibility of the XID mouse to *C. gattii*, which suggests that the presence of B-1 cells and anti-GXM antibodies is fundamental during the control of infection by *C. gattii*.

## Introduction

Cryptococcosis is an infectious disease caused by the fungal species *Cryptococcus neoformans* and *Cryptococcus gattii*. Both are globally distributed, although *C. gattii* is generally seen as a tropical or subtropical fungus^1–3^. Cryptococcosis affects mostly immunocompromised individuals causing severe lung disease and meningoencephalitis, with *C. gattii* being able to infect immunocompetent hosts^4,5^. During *C. neoformans* infection, it is possible to characterize the production of natural and specific IgM, induced by capsular components and by the presence of highly conserved microbial antigenic determinants, such as α- and β-glucans, which are present in its cell wall^6^. Glucuronoxylomannan (GXM) is the major component of the *Cryptococccus* capsule, representing 90% of the capsular mass^7,8^. This polysaccharide is also one of the most studied virulence factors of the yeast, a lot due to its immunomodulatory effects during infection^9–14^. Anti-GXM antibodies have been shown to be important opsonins in fighting infection by *Cryptococcus* spp., increasing the function of macrophages and promoting the death of yeasts^15^. In the context of humoral response, the production of antibodies limits the progression of cryptococcosis and helps the activation of the cellular response against the fungus^16^. Therefore, during fungal infection some effects have already been observed. For instance, the number of IgM^+^ B cells in the blood, which is lower in HIV-infected individuals who have developed *C. neoformans* infection than those who have not. In addition, HIV-infected individuals have lower levels of GXM-specific IgM, than non-HIV-infected individuals^17,18^. Furthermore, IgM deficiency in a murine model makes animals more susceptible to *C. neoformans* infection^19^. Very little is known about the role of humoral immunity during the *C. gattii* infection.

B-1 cells are a population of cells with an activity similar to cells of the innate immunity and can be divide between B-1a (CD5^+^) and B-1b (CD5^−^) subtypes^20^. They produce natural IgM without the need of antigen stimulation; therefore, they can be considered as a population inherently prepared to fight infections^21–23^. It has been reported that B-1a and B-1b populations produce natural antibodies against the capsular components of *Streptococcus pneumoniae* mitigating this pathogen infection^24^. In addition to humoral activity, B-1 lymphocytes are able to act innately by differentiating themselves into a mononuclear phagocyte population and fighting *C. neoformans* in a nitric oxide-dependent manner^25^. However, it has been shown in *Paracoccidioides brasiliensis* infection that B-1 cells secretes IL-10 and induces T regulatory cells activation facilitating the progression of the disease^26,27^. This contribution of IL-10 production by B-1 cells was also seen in *Leishmania* and *Francisella* infections^28–30^. Therefore, B-1 cells appear to differently commit their effector function in distinct infections. Furthermore, in the aspect of the production of natural antibodies, the role of IgM produced by B-1 cells appears to be related to the control of infection of *C. neoformans* by preventing their spread from the lungs to the brain and restricting the size of yeasts^31^. However, the role that B-1 cells play in controlling a *C. gattii* infection has not yet been evaluated.

In the present study, mice with X-linked immunodeficiency (XID) were used, these animals have a mutation in the gene that encodes the protein tyrosine kinase of Bruton (Btk), leading to a blockade in the B cells development, predominantly B-1 cells^32^. These cells reside mainly in the peritoneal and pleural cavities, secreting IgM and IgG2 in response to type 2 independent antigens (TI-2)^33^. Our model of chronic lung infection revealed that XID mice, compared to normal animals, had early mortality with significant weight loss, reduced serum GXM-specific IgM and IgG titer and increased blood and brain fungal load. In addition, there was a capsular size increase in *C. gattii* and the predominant presence of cytokines with the Th2 profile was observed in these animals. Thus, our data demonstrated that XID mice are more susceptible to infection by *C. gattii*, suggesting that the modulating effects mediated by B-1 cells with the production of anti-GXM antibodies and cytokines contribute to the protection against infection.

## Results

**Figure 1:**
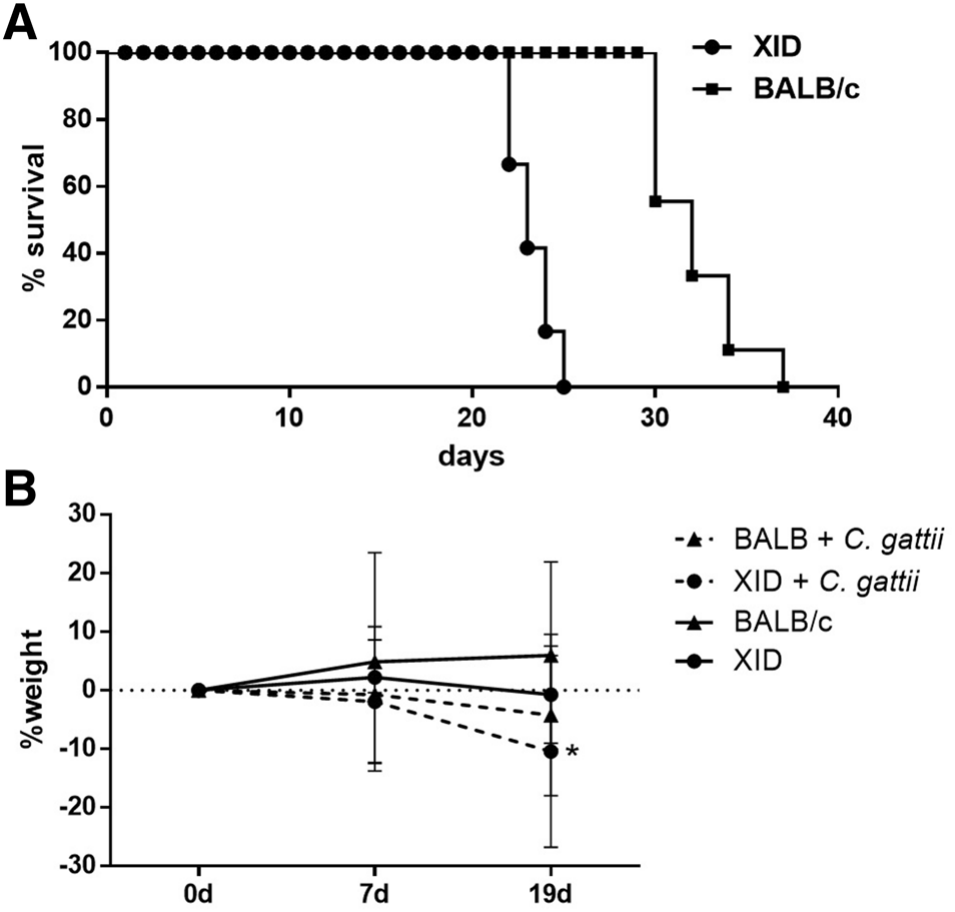
Evaluation of XID and BALB/c susceptibility to *C. gattii* infection. (**A**) Survival assay of XID (n = 10) and BALB/c (n = 10) mice infected with *Cryptococcus gattii* strain R265 belonging to serotype IIa. Time in days post-infection is shown on the x-axis. (**B**) The weight variation of the XID (n = 12) and BALB/c (n = 12), (infected or uninfected) mice was monitored during the infectious process, 0, 7 and 19 days of infection were the times stipulated for analysis. The graphs show the results of 2 independent experiments. (^###^p < 0.001) for comparative degree between strains. (*p < 0.05) for comparative degree between days 0 and 19 (0 d × 19 d).

Studies using B-1 cells of deficient mice (XID mice), suggested an important role for these cells during the immune responses against infections^29,30,34,35^. This led us to investigate if the B-1 cells would be implyed in the response against *C. gattii* infection on this animal model. First, XID and BALB/c mice were infected with 1 × 10^4^ yeasts of *C. gattii* intratracheally to assess survival and weight loss of the mice was also analyzed at days 0, 7 and 19 after infection. Our results showed that BALB/c and XID mice are susceptible to infection by *C. gattii*, but XID mice had a lower percentage of survival (Fig. 1A). In addition, the most evident weight loss was found in the XID mice with significant percentage at 19 days after infection (Fig. 1B). These data suggest that XID mice are more susceptible to *C. gattii* than BALB/c mice.

**Figure 2:**
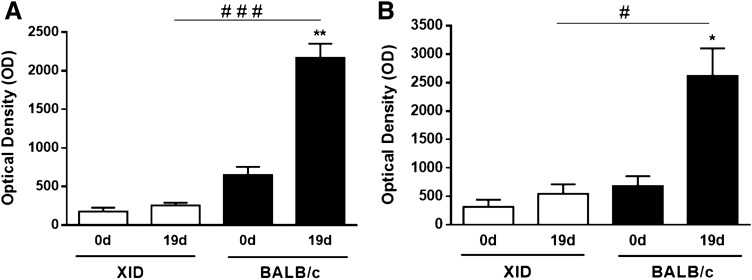
Dosage capsular anti-GXM antibodies from serum samples. (**A**) Immunoenzymatic assay (ELISA) with the serum of animals before infection (0 d) and infected (19 d), for the determination of IgM antibodies reactive against the glucuronoxylomannan polysaccharide (GXM) present in the capsule. (**B**) The same serum was subjected to analysis, by ELISA, for quantification of anti-GXM IgG antibodies. The graphs show the results of 2 independent experiments (n = 5). (**p < 0.01; *p < 0.05) for comparative degree between days 0 and 19 (0 d × 19 d), (^#^p < 0.05; ^###^p < 0.001) comparative degree between strains.

B-1 cells are considered a part of the innate immune system that have a carbohydrate-specific activity through a type 2 thymus-independent response, which then leads mainly in the natural IgM^36–39^. Thereby, considering that the XID mice had the greatest susceptibility to infection, we decided to assess whether the antibody production against the *C. neoformans* capsular polysaccharide GXM was established, compared to the non-XID model. For this, we performed the analysis of the production of specific antibodies against purified GXM on days 0 and 19 after infection. Our results showed that BALB/c mice produce specific IgM and IgG antibodies against GXM, while XID mice did not show significant antibody production (Fig. 2).

*Cryptococcus* can spread from the lungs by hematogenous route evading the local immune response. This allows the pathogen to cross the blood–brain barrier (BBB), causing the most aggressive form of the disease, the cryptococcal meningoencephalitis^40–42^.

**Figure 3:**
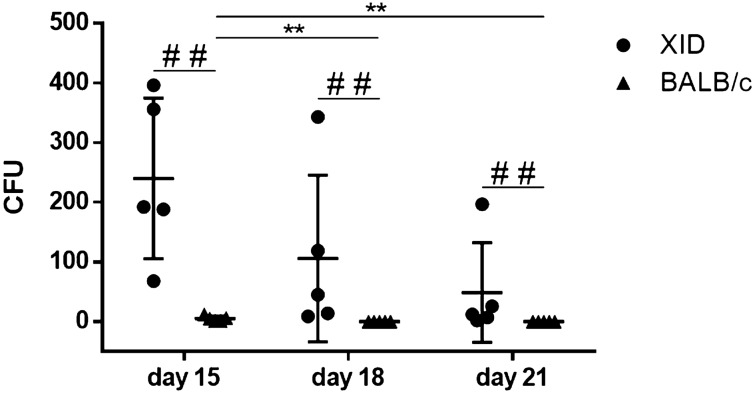
Investigation of the passage of *C. gattii* by the hematogenous route of XID and BALB/c mice. Counting of Colony Forming Units (CFU) of the peripheral blood of XID and BALB/c mice on days 15, 18, and 21 of the infectious course. Colony growth was not observed on days 3, 6, 9 and 12 (data not shown). The graphs show the results of 2 independent experiments (n = 5) (^###^p < 0.001).

We evaluated the presence of *C. gattii* in the hematogenous pathway of XID and BALB/c mice, analyzed the content of whole blood plated on sabouraud agar and observed only colony growth in XID mice on days 15, 18 and 21. BALB/c mice presented CFU count only 15 days after infection (Fig. 3). The amount of yeasts disseminated in the peripheral blood of BALB/c mice was lower than observed in XID mice, and these, despite the high parasitism evidenced, showed a reduction in the number of colonies over the days observed (Fig. 3).

**Figure 4:**
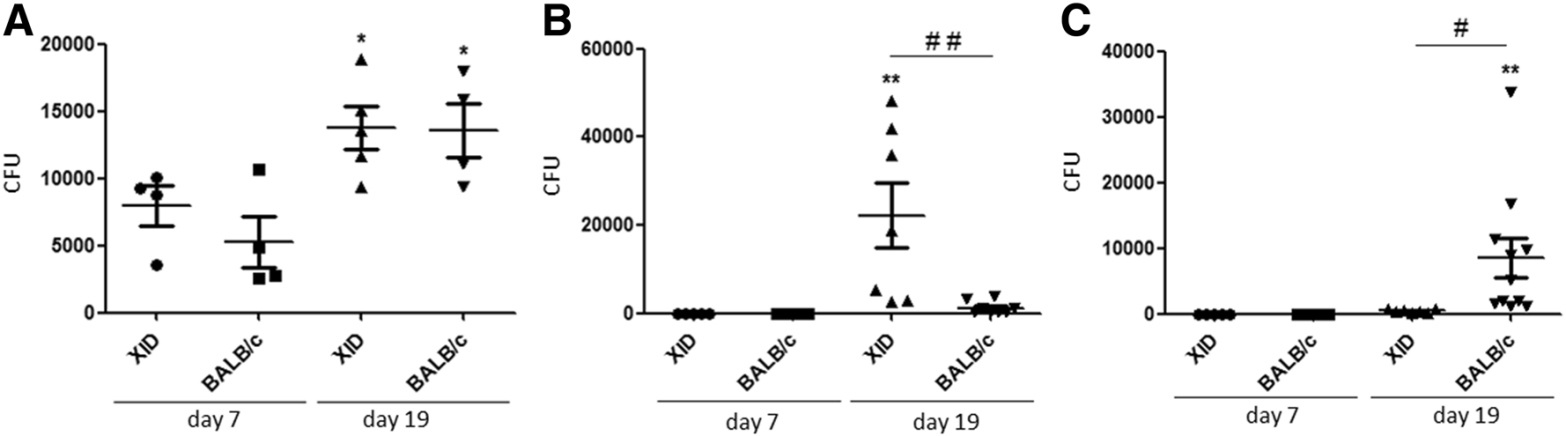
Analysis of fungal load in different target organs involved in cryptococcosis. (**A**) Analysis of the fungal load on days 7 and 19 of the bronchoalveolar lavage (BAL) of XID (n = 5) and BALB/c (n = 5) mice infected with *C. gattii*. (**B**) The brain of infected XID (n = 7) and BALB/c (n = 7) mice were also subjected to the counting of colony-forming units (CFU) at the same time. (**C**) Colony Forming Units (CFU) of the spleen were quantified under the same aspects (n = 11). Each symbol represents a value for an individual mouse, with the average indicated by the black line. The graphs show the results of 3 independent experiments. (*p < 0.05) for comparative degree between days 7 and 19 (7 d × 19 d), (^##^p < 0.01; ^#^p < 0.05) for comparative degree between strains.

In the next step we performed the CFU counts of the XID and BALB/c animals on days 7 and 19 of infection. We used the bronchoalveolar lavage, spleen and brain. Our results demonstrated that there was no difference in the growth of bronchoalveolar lavage colonies between XID and BALB/c mice on days 7 and 19 of infection (Fig. 4A). Remarkably, only the XID mice showed a high number of CFU in the brain on the 19th day of infection (Fig. 4B), while the spleen of BALB/c mice showed a higher number of CFU on the same time of infection (Fig. 4C). Similar results were not seen in the brain and spleen macerated on day 7 of infection (Fig. 4B,C). Previous results using in vitro models suggest that the initial site of cryptococcal entry into the brain is the BBB^43^. It has also been shown that *C. neoformans* yeast cells interact and cross the BBB using brain microvascular endothelial cells^41^. In an experimental infection study involving intravenous injection of *C. neoformans* in mice, it was demonstrated the first morphological evidence that *C. neoformans* enters the brain through BBB endothelial cells by a transcellular mechanism^41^.

**Figure 5:**
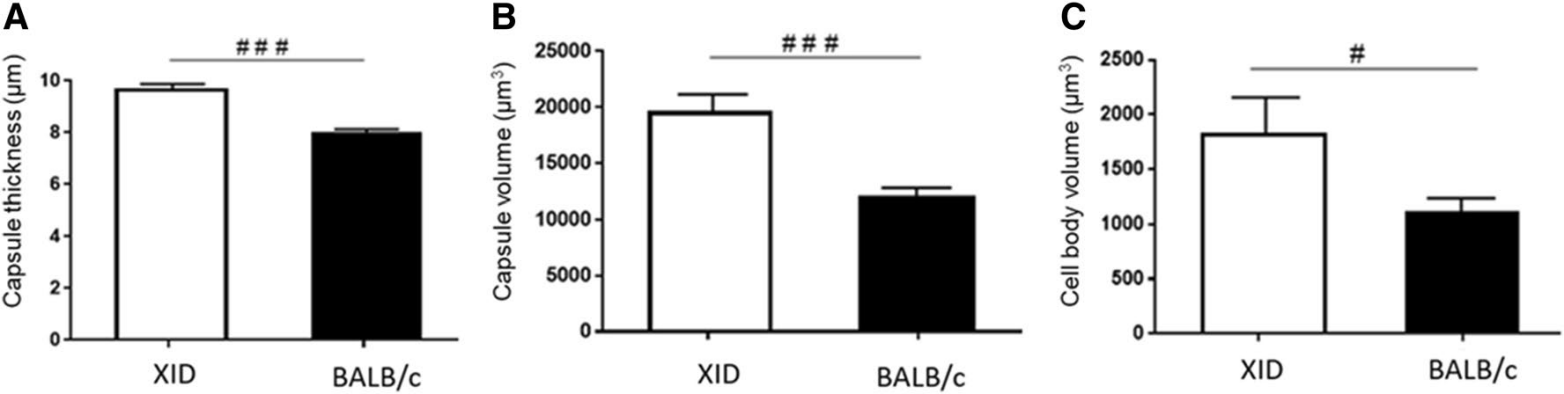
Analysis of *C. gattii* size in the BAL of XID and BALB/c mice. (**A**) Capsule thickness, (**B**) capsule volume and (**C**) *C. gattii* cell body volume in the bronchoalveolar lavage (BAL) of animals XID and BALB/c with 19 days of infection. Samples fixed with 4% formaldehyde, stained with India ink and analyzed under a microscope at 400 × magnification. The graphs show the results of 2 independent experiments (n = 5). (^###^p < 0.001; ^#^p < 0.05).

Depending on the host environment, *Cryptococcus* can undergo considerable morphological changes that will be essential to establish the infection^31^. Briefly, *Cryptococcus* changes shape and produces enlarged cells called giant cells or “titan cells”^44–47^. This phenomenon has been observed in different infection models^46,48–51^. The titan cells enlarge the cell body, ranging from 10 to 100 μm in diameter^52^. Some research groups described that these cells have a large and single vacuole and that, in addition to having a thick cell wall, they also have a dense polysaccharide capsule. The titan cells are highly resistant to oxidative stress and phagocytosis^53,54^. The presence of these giant cells increases the spread of the fungus, amplifying the cryptococcal virulence^55^. Therefore, to relate possible modifications in capsular dimensions with the spread of *C. gattii* in the XID mice, we measure the size changes of the yeasts. Our results showed that the yeasts of *C. gattii* obtained from the bronchoalveolar lavage (BAL) of XID mice on the 19th day of infection, showed an increase in thickness (Fig. 5A) and capsular volume (Fig. 5B). On the other hand, yeasts obtained from BAL from BALB/c mice, presented smaller dimensions. (Fig. 5A–C).

**Figure 6:**
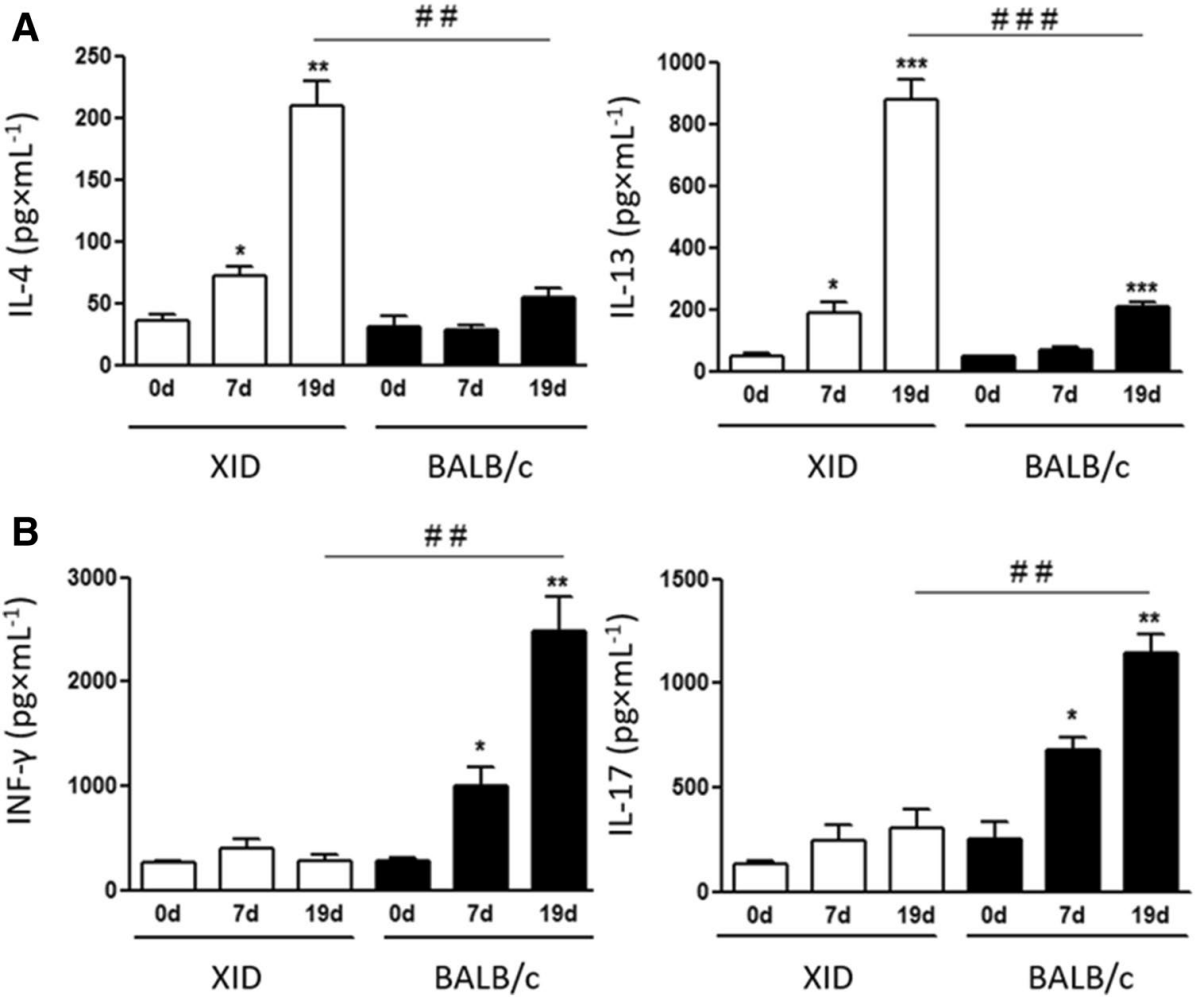
Cytokine dosage of bronchoalveolar lavage in XID and BALB/c mice. (**A**) Immunoenzymatic assay (ELISA) of the bronchoalveolar lavage (BAL) of XID and BALB/c animals before infection (0 d) and infected (7 d and 19d), for the measurement of cytokines IL-4 and IL-13 (**B**) and, IFN-γ and IL-17. The graphs show the results of 2 independent experiments (n = 5). (***p < 0.001; **p < 0.01; *p < 0.05) for comparative degree between days 0 and 7 (0 d × 7 d) or 0 and 19 (0 d × 19 d). (^###^p < 0.001; ^##^p < 0.01) for comparative degree between strains.

It has been reported that *C. gattii* dampens some immune cell functions through its polysaccharides content, mainly due the regulation of their cytokine profile^56–58^. Previous results indicate that infection by *C. gattii* induces the production of cytokines related to the Th1 profile more than other species of *Cryptococcus*, such as IL-1β, TNF-α and IL-6. It is also related to the induction of the Th17 profile with the production of the cytokines IL-17 and IL-22^59^. Another important fact that shows the regulation mediated by cytokines was the observation that a change to the profile of Th2 cytokines is associated with the worsening of *C. neoformans* infection^60^. Thus, in our model, we accessed the bronchoalveolar lavage levels of cytokines associated with different cellular profiles, IL-4 and IL-13 (Th2), INF-γ (Th1) and IL-17 (Th17) of XID and BALB/c mice before infection (day 0) and 7 and 9 days after infected with *C. gattii*. Our results showed an increase in cytokines IL-4 and IL-13 in the BAL of XID mice on the 7th and 19th day of infection (Fig. 6A). In contrast, the predominant cytokines in the BAL of BALB/c mice were IFN-γ and IL-17 (Fig. 6B), with a slight increase to IL-13 (Fig. 6A).

## Discussion

Cryptococcosis in its clinical aspect is characterized by causing severe damage to the lungs, and due to the tropism of yeast by the brain, infection can trigger meningoencephalitis in the most severe cases^61^. Nevertheless, in the context of the disease agents, there is a limitation in the availability of studies dealing with the pathology of *C. gattii* infection, with most studies focusing on *C. neoformans* species^4^. Furthermore, an important aspect of the response against the disease is the establishment of a humoral response against the fungus, and this can occur by the recognition of the major component of the *Cryptococcus* capsule, the polysaccharide GXM by the B cells, who ends up eliciting a strong antibody production response^62^. These antibodies are responsible for blocking the release of polysaccharide antigens and in assisting the disease resolution^12,63^. Incidentally, most of the IgM antibodies produced primarily in the early stages of an infection come from the population of B-1 cells, which not only contribute to humoral immunity by producing natural antibodies, but also have innate-like activity^21–23,25^.

Due to this scenario, the present study had intent to contribuit to the understanding of the immunopathology of the *C. gattii* infection, focusing mainly on the influence that the B-1 cell population has on the infection. Previously, a study using the XID mice model, deficient of B-1 cells population^32^, had already shown that this population of cells are involved in dealing *C. neoformans* infection^31^. In agreement, our results using *C. gattii* demonstrate that XID mice are more susceptible to *C. gattii* infection, revealed by the lower survival rate and lower weight score during the infection. Furthermore, the worsening of cryptococcosis is related to a weakened immunity by pre-existing conditions or co-infections. Showing that the malfunction of determinants of the immune system corroborate for a higher mortality and susceptibility to cryptococcosis^64^. Therefore, the absence of the B-1 cell component in the XID mice characterizes the loss of cells that are very important for infection resolution^24,25,38,65^, which justify the worsening of the clinical score that has been observed previously using *C. neoforomans*^66^, and now with *C. gattii*. We also found that the XID animals still had an extremely deficient serum level of IgM and IgG anti-GXM antibodies, suggesting that antibodies play an important role in infection and act by limiting the spread of yeast. The phenotype of the infected XID mice in this study had similar aspects to that previously reported with knockout for IgM, who describes the occurrence of an enhanced susceptibility to *C. neoformans* infection when limiting the production of IgM^19^. Moreover, our data also highlight an important role of the B-1 cells during chronic infection, as seen by the higher IgM antibody titer compared with the antigen-specific IgG antibody titer between the XID and BALB/c mouse. B-1 cells are involved in fighting *C. neoformans* infection, also due to their production of natural IgM antibodies against the yeast capsular content, while B-2 cells could act in the production of antigen-specific IgG antibodies^22,67^, so B-1 cells ends up being responsible for a large part of IgM in the early stages of *C. gattii*, and therefore, as we reveled, this amount was compromised in the XID mice.

The lower survival of XID in relation to the control group BALB/c may reflect the differences in IgM found between these two groups. Our results indicate a greater magnitude of serum IgM indexes during the establishment of infection in the BALB/c group. It is possible that such differences may influence the onset of *C. gattii* colonization, considering that our findings demonstrate higher CFU indices in XID in the different tissues analyzed. The low levels of IgM induced at the onset of infection characteristic of the XID animals may be correlated with the increased susceptibility of these animals in relation to the controls. However, the immune responses induced by IgM are decisive during the control phase of the colonization of the pathogen in the host, and are therefore not critical in the development of protection, a fact that would explain a delay in the infection of wild animals, and a consequent increase in the survival curve.

Although the different levels of IgG antibodies between the XID and BALB/c mice were lower, we did not deny a possible contribution of conventional B-2 cells in our model. In support of this idea, antibodies derived from both B-1 and B-2 cells are important against cryptococcal and other infections^68–71^. However, regardless of the exact source of B cells, our data suggest that antibody deficiency impairs containment of *C. gattii* in the lungs.

The role of B-1 lymphocytes in antibody production has been characterized. However, these cells also have other characteristics that can play important mechanisms in protecting against different pathogens, and one of these mechanisms is their ability to differentiate into phagocytes called B-1 cell-derived phagocytes (B-1CDP)^30,34,72–74^. It was also shown that B-1 cells migrate to the inflammatory/infectious focus and differentiate into macrophage-like cells^75^. Ghosn and colleagues^25^ demonstrated that B-1CDP cells are able to internalize *C. neoformans* and this process is mediated by the complement receptor 3^25^. It was also characterized by the same group that B-1CDP phagocytes had fungicidal activity mediated by the production of nitric oxide (NO) and the antigens processed by these cells were presented through the expression of molecules of the major histocompatibility complex type II (MHC II), resulting in considerable production of inflammatory mediators^25^. Based on this information, it is possible to relate the deficiency in the production of B-1 lymphocytes and the consequent decrease in the differentiation of B-1CDP cells in XID mice thus compromising the link between innate and adaptive immunity in experimental infection by *C. gattii*.

Despite the problems observed in the absence of Btk-mediated signaling in the functional activities of B lymphocytes, the absence of this signaling can also affect the activity of myeloid cells. Deficiency in Btk-mediated signaling is related to failures in the production of microbicidal factors in neutrophils and macrophages^35,76–78^. The COVID-19 pandemic has driven the scientific community to search for drugs that could reduce the viral load or negatively modulate the cytokine storm seen in the severe conditions of COVID-19. Thus, it was described that the use of inhibitors of Btk-mediated activation could be evaluated in these patients^79^. On the other hand, Honda and colleagues described contradictory results in which it was shown that neutrophils from XID mice were able to produce ROS in response to activation of the Toll-like receptor (TLR)^80^. According to this information, a trend towards increased transcription of inducible nitric oxide synthase in the lungs of *C. neoformans*-infected XID mice has been evidenced^31^. Therefore, the importance of Btk-mediated signaling in myeloid lineage cells remains unclear.

We consistently found a higher fungal burden in the lungs on the later days of infection in both XID and BALB/c groups, but surprisingly when we evaluated the fungal load in the brain, only the XID group had shown higher values of CFU. Moreover, this profile of fungal load had already been seen on the literature using *C. neoformans*^31^. This indicates that possibly, *C. gattii* yeasts where preferably migrating to the CNS in the mice group absent of B-1 cells, whereas in the BALB/c group, the higher fungal load was only found on spleen. Although there have been no studies on the interference of B-1 cells in brain infection by *C. gattii*, B cell-dependent protection of brain infection by *C. neoformans* has been described, and most interestingly, it is that this protection was evaluated in a Rag^−/−^ model, showing that this effect seems to be independent of T cells^81,82^, and in consensus with our data, a possible parallel could be made in the *C. gattii* infection model, although further studies in this regard need to be done.

Thus, this consistently leads us to discuss whether the lack of humoral response by B-1 cells could contribute in any way to a greater escape of yeast from the lungs to other tissues and, therefore, to the bloodstream. Although we have not shown whether *C. gattii* cells were inside monocytes in the bloodstream, the Trojan horse mechanism is the main hypothesis believed for the yeast to reach the CNS within phagocytes^42,43^. For instance, a study using a murine model revealed that 1.7% to 2% of yeast cells deposited in the bloodstream, through injection into the tail vein, remained after 30 min of infection^41^, showing that these yeasts survive in circulating blood, despite the host defense system efficiently cleared part of the organisms from the bloodstream. With the analysis of *C. gattii* CFU from XID mice’s peripheral blood, our data demonstrated an increased fungal load in hematogenous pathway during infection period, in comparison to BALB/c mice.

Our data demonstrated that *C. gattii* CFU counts in the peripheral blood of XID mice, during the period of infection, presented an increased fungal load in the hematogenous pathway when compared to BALB/c mice. An interesting data we obtained was that when analyzing the amount of CFU in the bloodstream of XID mice over the 15th, 18th and 21st days after infection, there was a gradual reduction in the fungal load that can be explained by the displacement of the yeasts to other organs, such as the brain tissue. Moreover, it has already been shown that the loss of an immune function, as in immunosuppressed patients, can contribute to a greater blood fungal load during *C. neoformans* infection^83^, therefore the loss of B-1 activity in XID model is comparatively similar during *C. gattii* infection in our study.

An important feature that contributes to the infectious process of cryptococcosis is the increase in the yeast’s polysaccharide capsule. This process is believed to provide an important advantage for the pathogen, since the capsule promotes resistance to phagocytosis and oxidative stress^53^. We here observed that the size of *C. gattii* yeasts in the bronchoalveolar lavage of XID mice consisted of yeasts with larger cell bodies and capsules sizes compared to BALB/c mice. This profile of capsular and body enlargement had already been seen on a previous work with *C. neoformans*^31^. Still, there are reports in the literature of formation of giant cells in the lung of mice with cryptococcosis. These giant cells have cell body volume above 10 μm and are normally called titan cells^84^.

Titan cells are described as yeasts that are abnormal in size and have a strong immunomodulatory role^44,55^. Thus, it is believed that they are cells present in different tissues where the damage caused by cryptococcal infection is intense^47^. The size protects the fungus from capture by alveolar phagocytes, as proposed for *C. neoformans*^25^. Phagocytosis by alveolar macrophages is the most important response to *Cryptococcus* spp. in the lungs and the titan cells have been show to be vital for the establishment of early lung infection^54^. In addition, titan cells are resistant to free radicals of oxygen and nitrogen from the host's immune cells and have a thicker polysaccharide capsule and cell wall that can act as defensive barriers^55^. Recently, our group was the first to demonstrate the presence of titan cells in the lung of mice infected with *C. gatti*^85^.

So far, the titan cells formation seems to be a unique feature to *Cryptococcus* species complex that allows protection from phagocytosis^52,54^. This process of capsule enlargement and titan cells formation reshapes the cell wall and capsule composition during infection and enhances the virulence of *Cryptococcus* spp.^55,86^. The formation of titan cells can be attributed to several factors during infection, such as iron limitation, in which has already been described as a limiting condition for the spread of *C. gattii*. Therefore, the yeast has the potential to modify its metabolism in these conditions to produce titan cells and evade the immune response^87,88^. In this regard, it has already been shown, that the formation of titan cells in *C. neoformans* depends, not only on microbe factors, but also on the host environment conditions when considering different mice model^89^, as in the XID model. Titan cells formation is also related with the induction of a Th2 cytokine profile and a greater presence of specific IgM for the *C. neoformans* capsular polysaccharides^89^. We observed in XID mice a prevalence of Th2 profile cytokines production, like IL-4 and a lower production of GXM-specific IgM, since only BALB/c mice, that have B-1 cells, they presented the largest amount of IgM production. Indeed, XID mice had lower cytokines levels associated with the Th1 and Th17 profile during *C. gattii* infection, similar to that previously described by our group with a TLR9^−^/^−^ mice model, where the most interesting thing aspect found was that this cytokine profile was associated with a greater formation of titan cells and a greater brain fungal load^85^. In this regard, it had already been described that IgM production inhibits titan cell formation of *C. neoformans*^67^, therefore, B-1 cell humoral response to *C. gattii* cryptococcosis appears to be essential to prevent yeast cell enlargement and by that, possibly assuring the limitation of yeast dissemination to the brain.

In summary, the XID mice showed significantly marked infection in the brain with reduced survival. This scenario may be the result of the drastic reduction in the production of specific antibodies against *C. gattii* and production of cytokines of the Th1 and Th17 profile. Our results together point to the crucial role of B-1 cells in preventing a marked spread of *C. gattii* from the lungs to the brain in a model of chronic lung infection.

## Methods

### *Cryptococcus* strain

*Cryptococcus gattii*, serotype B, strains R265, hypervirulent, molecular type VGIIa, with mating type α. Kindly provided by Prof. Leonardo Nimrichter, head of the microbial biochemistry laboratory at the Paulo Góes Institute of Microbiology at the Federal University of Rio de Janeiro (UFRJ). The yeasts were kept in an incubator at 28 °C, 5% CO_2_, in minimal medium, containing: 2.7 g × L^−1^ glucose (C_6_H_12_O_6_); 2.5 g × L^−1^ of magnesium sulfate hephidrated (MgSO_4_ × 7H_2_O); 4.0 g × L^−1^ of monobasic potassium phosphate (KH_2_PO_4_); 10 g × L^−1^ glycine (C_2_H_5_NO_2_); 0.001 g × L^−1^ thiamine (C_12_H_17_N_4_OS).

### Inoculum preparation

After raising the fungus 48 h before use, the sample was led to centrifugation at 4985 G for 3 min (Micro High Speed Refrigerated Centrifuge VS-15000 CFN II CE), and then it was resuspended in 1 mL of sterile PBS (phosphate buffered saline) and centrifugated again in the same conditions (for 2 ×). Then the precipitated was resuspended in 1 mL of sterile PBS (phosphate buffered saline), diluted at 1:1000 ratio in sterile PBS (phosphate buffered saline) and it was counted the viable yeasts for the Neubauer chamber experiment (Microscope Nikon Eclipse E2000). Inoculum was containing 1 × 10^4^ fungal cells per animal.

### Mice

Isogenic female mice of BALB/c-XID lineage and BALB/c controls, female, aged 8 to 12 weeks, weighing an average of 20.5 and 24.5 g, respectively were used in this study. The XID mice were kindly donated by the Laboratório de Imunomodulação, Centro de Ciências da Saúde, Instituto de Biofísica Carlos Chagas Filho, Universidade Federal do Rio de Janeiro, RJ, Brazil, and the BALB/c lineage was kindly donated by the Instituto de Veterinária, Departamento de Microbiologia e Imunologia Veterinária, Universidade Federal Rural do Rio de Janeiro, RJ, Brazil. The animals were maintained in sterile (grouped) cages, under standardized conditions of temperature (22–23 °C) and light (cycles of 12 h of light and 12 h of dark), commercial feed and drinking water provided ad libitum^85^. The use of the animals in this study was approved by the Ethics Committee on the Use of Animals (CEUA) at UFRJ (Nº: 078/19). The mice were sacrificed according to the criteria approved by CEUA at the time of the study (2019/2020). All animal work was performed in accordance with Animal Research: Reporting of In Vivo Experiments (ARRIVE) guidelines and regulations and all methods were carried out in accordance with relevant guidelines and regulations.

### Anesthesia and analgesia

Before the surgical procedure, anesthesia and analgesia of the animals were performed intraperitoneally with xylazine 0.2 mg × mL^−1^ (10 mg × kg^−1^) and ketamine 20 mg × mL^−1^ (100 mg × kg^−1^) in each animal.

### Inoculum and intratracheal infection

An intratracheal injection of 1 × 10^4^ fungal cells per animal with 30 μL per mouse inoculum was performed in tested animals, whereas in control animals the same procedure was performed by injecting 30 μL of sterile PBS per mouse.

### Serum aquisition

Blood samples were collected from uninfected XID and BALB/c mice (NI), infected for 7 days with *C. gattii* (7d) or infected for 19 days with *C. gattii* (19d). Mice were anesthetized and blood collected by retro-orbital puncture. Serum samples were obtained after allowing blood samples to clot for 30 min and be centrifuged at 915 G for 6 min (Micro High Speed Refrigerated Centrifuge VS-15000 CFN II CE). Collected serum samples were stored at − 80 °C until further analysis.

### Bronchoalveolar lavage aquisition (BAL)

The bronchoalveolar lavage procedure was performed with syringes containing 1000 μL of sterile PBS (phosphate buffered saline), in infected or non-infected mice, and the samples were kept on ice until further analysis.

### Animal survival and weighing variation

After an intratracheal injection of 1 × 10^4^ yeasts per animal (n = 10), mice were observed during all the days of infection and the deaths were recorded for survival analysis. The animals (n = 12) were weighed before infection (day 0) and during infection (days 7 and 19), with the respective averages obtained.

### IgM and IgG anti-GXM antibodies dosage

The serum of 5 infected mice (day 19) and 5 not infected mice (day 0), was submitted to the measurement of antigen-specific antibodies. For that, serum antibodies were determined by an immunoenzymatic assay (ELISA). 96-well plates were coated with 10 μg × mL^−1^ of purified GXM from *C. neoformans*. Isolation and purification procedures were adapted from protocols mentioned before^9–13^. The mice serum was diluted in 1:3 ratio within a container with PBS and 1% of bovine serum albumin (1% BSA-PBS). Afterwards, rabbit anti-IgM or anti-IgG antibodies were added. Marking was made with anti-IgM or anti-IgG and alkaline phosphatase at a 1:2500 ratio following the protocol of Szymczak and collaborators (2013). The analysis was made by an ELISA reader (VERSAmax Tunable microplate reader—T4.0A) at 450 nm.

### Fungal load

Brain (7 animals per group) and spleen (11 animals per group) were collected on days 7 and 19 of infection, 50% of their volume was macerated and diluted in 1 mL of sterile PBS (phosphate buffered saline) in a petri dish, the after, it was poured 10 μL in a new petri dish containing sabouraud agar (1:100 ratio) (20 g × L^−1^ glucose (C_6_H_12_O_6_); 10 g × L^−1^ peptone; 40 g × L^−1^ sabouroud agar—Sigma, USA). With bronchoalveolar lavage and peripheral blood from infected mice (5 animals per group), it was poured 10 μL of the content into a new petri dishe containing sabouraud agar (1:100 ratio for BAL and blood without dilution). Then, it was left in an oven at 37 °C, 5% CO_2_. Plaque analysis was performed after 72 h by counting colony-forming units (CFU). Photographs were taken as representative of the data.

### *C. gattii* capsule size and cellular body measurement

For measuring yeast diameter, suspensions were prepared from bronchoalveolar lavage from infected XID and BALB/c mice for 19 days (5 animals per group). The samples were initially centrifuged at 4985 G for 6 min at 4 °C (Micro High Speed Refrigerated Centrifuge VS-15000 CFN II CE), the supernatant was discarded, and the precipitate resuspended with 500 μL of 4% formaldehyde. After 30 min, it was again centrifuged at 4985 G for 6 min (Micro High Speed Refrigerated Centrifuge VS-15000 CFN II CE), supernatant was discarded, and precipitate resuspended in 500 μL of PBS (phosphate buffered saline). The visualization occurred after gently mixing, to avoid the formation of foam, 10 μL of the suspension of cells with 5 μL of Indian ink and examined under a microscope (Observer Z.1—Zeiss), with a magnification of 400 ×. India ink particles are repulsed by the *C. gattii* capsule, which is easily observed microscopically^90^. Whole cell (Wc) and cell body (Cb) measurements were taken for a minimum of 100 cells per experimental group. After the measurements, the data were processed to obtain the values of capsule thickness, capsule volume and cell body volume. To obtain capsule thickness values, the following calculation was used: Capsule thickness = (Wc-Cb)/2 and Cell body volume = 4 × (π × r^3^)/3. To calculate the capsule volume, the total cell volume was calculated, and the volume of the cell body was subtracted from it.

### Cytokine dosage

Bronchoalveolar lavage (BAL) was obtained on days 7 and 19 of infection (n = 5). The samples were centrifuged at 260 G for 6 min (Micro High Speed Refrigerated Centrifuge VS-15000 CFN II CE) and the supernatant was centrifuged again at 4985 G for 6 min. The samples were kept at -80 °C until dosing. Serum cytokines were measured by the capture method, following the company's protocol (R&D Systems, Minneapolis, MN). The 96-well plate was sensitized with standard antibodies to IL-13, IL-4, IL-17 and INF-γ for 24 h, followed by blocking the wells in favor of preventing unwanted reactions, then after, 25 μL of the samples from the BAL with recombinant antibodies were incubated. After washing, detection antibodies were added and the reaction was developed with streptavidin–phycoerythrin. The analysis was made by an ELISA reader (VERSAmax Tunable microplate reader—T4.0A) at 450 nm.

### Statistical analysis

Statistical analysis was performed using the GraphPad Prism 6.0 program, using the Student T test for comparison between two groups and Log-rank test for comparison between two groups for the survival curve. For Fig. 3 was performed the Wilcoxon Rank Sum Test (Mann–Whitney *U*) obtaining the StandartDeviation (SD) values for each statistical analysis. Values of p < 0.05 indicate statistical significance, with ***p < 0.001; **p < 0.01; *p < 0.05 (or ^###^p < 0.001; ^##^p < 0.01; ^#^p < 0.05).

## References

[1] Garelnabi M, May RC (2018). Variability in innate host immune responses to cryptococcosis. Mem. Inst. Oswaldo Cruz.

[2] May RC, Stone NR, Wiesner DL, Bicanic T, Nielsen K (2016). Cryptococcus: From environmental saprophyte to global pathogen. Nat. Rev. Microbiol..

[3] Maziarz EK, Perfect JR (2016). Cryptococcosis. Infect. Dis. Clin. N. Am..

[4] Chen SC, Meyer W, Sorrell TC (2014). *Cryptococcus gattii* infections. Clin. Microbio.l Rev..

[5] Springer DJ (2014). *Cryptococcus gattii* VGIII isolates causing infections in HIV/AIDS patients in Southern California: Identification of the local environmental source as arboreal. PLoS Pathog..

[6] Rapaka RR (2010). Conserved natural IgM antibodies mediate innate and adaptive immunity against the opportunistic fungus Pneumocystis murina. J Exp Med.

[7] Cherniak R, O'Neill EB, Sheng S (1998). Assimilation of xylose, mannose, and mannitol for synthesis of glucuronoxylomannan of Cryptococcus neoformans determined by 13C nuclear magnetic resonance spectroscopy. Infect Immun.

[8] Decote-Ricardo D (2019). Immunomodulatory role of capsular polysaccharides constituents of cryptococcus neoformans. Front. Med..

[9] LaRocque-de-Freitas IF (2018). Involvement of the capsular GalXM-induced IL-17 cytokine in the control of *Cryptococcus neoformans* infection. Sci. Rep..

[10] Oliveira DL (2010). Extracellular vesicles from Cryptococcus neoformans modulate macrophage functions. Infect. Immun..

[11] Rocha JD (2015). Capsular polysaccharides from *Cryptococcus neoformans* modulate production of neutrophil extracellular traps (NETs) by human neutrophils. Sci. Rep..

[12] Vecchiarelli A, Casadevall A (1998). Antibody-mediated effects against Cryptococcus neoformans: evidence for interdependency and collaboration between humoral and cellular immunity. Res. Immunol..

[13] Villena SN (2008). Capsular polysaccharides galactoxylomannan and glucuronoxylomannan from *Cryptococcus neoformans* induce macrophage apoptosis mediated by Fas ligand. Cell Microbiol..

[14] Walenkamp AM (1999). Cryptococcus neoformans and its cell wall components induce similar cytokine profiles in human peripheral blood mononuclear cells despite differences in structure. FEMS Immunol. Med. Microbiol..

[15] Mukherjee S, Lee SC, Casadevall A (1995). Antibodies to Cryptococcus neoformans glucuronoxylomannan enhance antifungal activity of murine macrophages. Infect. Immun..

[16] Feldmesser M, Mednick A, Casadevall A (2002). Antibody-mediated protection in murine *Cryptococcus neoformans* infection is associated with pleotrophic effects on cytokine and leukocyte responses. Infect. Immun..

[17] Pirofski L, Lui R, DeShaw M, Kressel AB, Zhong Z (1995). Analysis of human monoclonal antibodies elicited by vaccination with a *Cryptococcus neoformans* glucuronoxylomannan capsular polysaccharide vaccine. Infect. Immun..

[18] Subramaniam K (2009). IgM(+) memory B cell expression predicts HIV-associated cryptococcosis status. J. Infect. Dis..

[19] Subramaniam KS (2010). The absence of serum IgM enhances the susceptibility of mice to pulmonary challenge with *Cryptococcus neoformans*. J. Immunol..

[20] Kantor AB, Stall AM, Adams S, Herzenberg LA, Herzenberg LA (1992). Differential development of progenitor activity for three B-cell lineages. Proc. Natl. Acad. Sci. USA.

[21] Baumgarth N (2011). The double life of a B-1 cell: Self-reactivity selects for protective effector functions. Nat. Rev. Immunol..

[22] Rohatgi S, Pirofski LA (2012). Molecular characterization of the early B cell response to pulmonary Cryptococcus neoformans infection. J. Immunol..

[23] Stall AM, Adams S, Herzenberg LA, Kantor AB (1992). Characteristics and development of the murine B-1b (Ly-1 B sister) cell population. Ann. N. Y. Acad. Sci..

[24] Haas KM, Poe JC, Steeber DA, Tedder TF (2005). B-1a and B-1b cells exhibit distinct developmental requirements and have unique functional roles in innate and adaptive immunity to *S. pneumoniae*. Immunity.

[25] Ghosn EE, Russo M, Almeida SR (2006). Nitric oxide-dependent killing of *Cryptococcus neoformans* by B-1-derived mononuclear phagocyte. J. Leukoc. Biol..

[26] Noal V, Santos S, Ferreira KS, Almeida SR (2016). Infection with Paracoccidioides brasiliensis induces B-1 cell migration and activation of regulatory T cells. Microbes Infect..

[27] Popi AF, Godoy LC, Xander P, Lopes JD, Mariano M (2008). B-1 cells facilitate *Paracoccidioides brasiliensis* infection in mice via IL-10 secretion. Microbes Infect..

[28] Arcanjo AF (2017). B-1 cells modulate the murine macrophage response to *Leishmania major* infection. World J. Biol. Chem..

[29] Crane DD, Griffin AJ, Wehrly TD, Bosio CM (2013). B1a cells enhance susceptibility to infection with virulent *Francisella tularensis* via modulation of NK/NKT cell responses. J. Immunol..

[30] Firmino-Cruz L (2019). How to B(e)-1 important cell during leishmania infection. Front. Cell Infect. Microbiol..

[31] Szymczak WA (2013). X-linked immunodeficient mice exhibit enhanced susceptibility to *Cryptococcus neoformans* Infection. MBio.

[32] Rawlings DJ (1993). Mutation of unique region of Bruton's tyrosine kinase in immunodeficient XID mice. Science.

[33] Khan WN (1995). Defective B cell development and function in Btk-deficient mice. Immunity.

[34] da Rocha R (2019). B-1 cells may drive macrophages susceptibility to trypanosoma cruzi infection. Front. Microbiol..

[35] O'Riordan CE (2020). X-linked immunodeficient mice with no functional Bruton's tyrosine kinase are protected from sepsis-induced multiple organ failure. Front. Immunol..

[36] Baumgarth N (2017). A Hard(y) Look at B-1 cell development and function. J. Immunol..

[37] Iseki M (2004). Increased numbers of B-1 cells and enhanced responses against TI-2 antigen in mice lacking APS, an adaptor molecule containing PH and SH2 domains. Mol. Cell Biol..

[38] Smith FL, Baumgarth N (2019). B-1 cell responses to infections. Curr. Opin. Immunol..

[39] Sundstrom JB, Cherniak R (1992). The glucuronoxylomannan of *Cryptococcus neoformans* serotype A is a type 2 T-cell-independent antigen. Infect. Immun..

[40] Beardsley J, Sorrell TC, Chen SC (2019). Central nervous system cryptococcal infections in non-HIV infected patients. J. Fungi.

[41] Chang YC (2004). Cryptococcal yeast cells invade the central nervous system via transcellular penetration of the blood-brain barrier. Infect. Immun..

[42] Charlier C (2009). Evidence of a role for monocytes in dissemination and brain invasion by *Cryptococcus neoformans*. Infect. Immun..

[43] Sorrell TC (2016). Cryptococcal transmigration across a model brain blood-barrier: Evidence of the Trojan horse mechanism and differences between *Cryptococcus neoformans* var. grubii strain H99 and *Cryptococcus gattii* strain R265. Microbes Infect..

[44] Feldmesser M, Kress Y, Casadevall A (2001). Dynamic changes in the morphology of *Cryptococcus neoformans* during murine pulmonary infection. Microbiology.

[45] Frases S (2009). Capsule of *Cryptococcus neoformans* grows by enlargement of polysaccharide molecules. Proc. Natl. Acad. Sci. USA.

[46] Zaragoza O (2009). The capsule of the fungal pathogen *Cryptococcus neoformans*. Adv. Appl. Microbiol..

[47] Trevijano-Contador N, Rossi SA, Alves E, Landin-Ferreiroa S, Zaragoza O (2017). Capsule enlargement in *Cryptococcus neoformans* is dependent on mitochondrial activity. Front. Microbiol..

[48] Love GL, Boyd GD, Greer DL (1985). Large *Cryptococcus neoformans* isolated from brain abscess. J. Clin. Microbiol..

[49] Okagaki LH (2010). Cryptococcal cell morphology affects host cell interactions and pathogenicity. PLoS Pathog..

[50] Garcia-Rodas R, Casadevall A, Rodriguez-Tudela JL, Cuenca-Estrella M, Zaragoza O (2011). *Cryptococcus neoformans* capsular enlargement and cellular gigantism during *Galleria mellonella* infection. PLoS ONE.

[51] Li Z, Nielsen K (2017). Morphology changes in human fungal pathogens upon interaction with the host. J. Fungi.

[52] Dylag M, Colon-Reyes RJ, Kozubowski L (2020). Titan cell formation is unique to *Cryptococcus species* complex. Virulence.

[53] Zaragoza O (2008). Capsule enlargement in *Cryptococcus neoformans* confers resistance to oxidative stress suggesting a mechanism for intracellular survival. Cell. Microbiol..

[54] Okagaki LH, Nielsen K (2012). Titan cells confer protection from phagocytosis in *Cryptococcus neoformans* infections. Eukaryot Cell.

[55] Crabtree JN (2012). Titan cell production enhances the virulence of *Cryptococcus neoformans*. Infect. Immun..

[56] Angkasekwinai P (2014). *Cryptococcus gattii* infection dampens Th1 and Th17 responses by attenuating dendritic cell function and pulmonary chemokine expression in the immunocompetent hosts. Infect. Immun..

[57] Huston SM (2016). *Cryptococcus gattii* capsule blocks surface recognition required for dendritic cell maturation independent of internalization and antigen processing. J. Immunol..

[58] Urai M (2015). Evasion of innate immune responses by the highly virulent *Cryptococcus gattii* by altering capsule glucuronoxylomannan structure. Front. Cell Infect. Microbiol..

[59] Schoffelen T (2013). *Cryptococcus gattii* induces a cytokine pattern that is distinct from other cryptococcal species. PLoS ONE.

[60] Leopold Wager CM, Hole CR, Wozniak KL, Olszewski MA, Wormley FL (2014). STAT1 signaling is essential for protection against Cryptococcus neoformans infection in mice. J. Immunol..

[61] Zaragoza O (2019). Basic principles of the virulence of *Cryptococcus*. Virulence.

[62] Mukherjee J, Casadevall A, Scharff MD (1993). Molecular characterization of the humoral responses to *Cryptococcus neoformans* infection and glucuronoxylomannan-tetanus toxoid conjugate immunization. J. Exp. Med..

[63] Martinez LR, Moussai D, Casadevall A (2004). Antibody to *Cryptococcus neoformans* glucuronoxylomannan inhibits the release of capsular antigen. Infect. Immun..

[64] Joao I, Bujdakova H, Jordao L (2020). Opportunist coinfections by nontuberculous mycobacteria and fungi in immunocompromised patients. Antibiotics.

[65] Chin SS, Chorro L, Chan J, Lauvau G (2019). Splenic Innate B1 B cell plasmablasts produce sustained granulocyte-macrophage colony-stimulating factor and interleukin-3 cytokines during murine malaria infections. Infect. Immun..

[66] Marquis G, Montplaisir S, Pelletier M, Mousseau S, Auger P (1985). Genetic resistance to murine cryptococcosis: Increased susceptibility in the CBA/N XID mutant strain of mice. Infect. Immun..

[67] Trevijano-Contador N (2020). Human IgM inhibits the formation of titan-like cells in *Cryptococcus neoformans*. Infect. Immun..

[68] Baumgarth N (2000). B-1 and B-2 cell-derived immunoglobulin M antibodies are nonredundant components of the protective response to influenza virus infection. J. Exp. Med..

[69] Torosantucci A (2009). Protection by anti-beta-glucan antibodies is associated with restricted beta-1,3 glucan binding specificity and inhibition of fungal growth and adherence. PLoS ONE.

[70] Yuan RR (1998). Isotype switching increases efficacy of antibody protection against *Cryptococcus neoformans* infection in mice. Infect. Immun..

[71] Zebedee SL (1994). Mouse-human immunoglobulin G1 chimeric antibodies with activities against *Cryptococcus neoformans*. Antimicrob. Agents Chemother..

[72] Arcanjo AF (2015). The PGE2/IL-10 axis determines susceptibility of B-1 cell-derived phagocytes (B-1CDP) to leishmania major infection. PLoS ONE.

[73] Oliveira VC (2018). Alteration in Ikaros expression promotes B-1 cell differentiation into phagocytes. Immunobiology.

[74] Popi AF, Zamboni DS, Mortara RA, Mariano M (2009). Microbicidal property of B1 cell derived mononuclear phagocyte. Immunobiology.

[75] Almeida SR (2001). Mouse B-1 cell-derived mononuclear phagocyte, a novel cellular component of acute non-specific inflammatory exudate. Int. Immunol..

[76] Mangla A (2004). Pleiotropic consequences of Bruton tyrosine kinase deficiency in myeloid lineages lead to poor inflammatory responses. Blood.

[77] Weber ANR (2017). Bruton's tyrosine kinase: An emerging key player in innate immunity. Front. Immunol..

[78] Mukhopadhyay S (2002). Macrophage effector functions controlled by Bruton's tyrosine kinase are more crucial than the cytokine balance of T cell responses for microfilarial clearance. J. Immunol..

[79] Roschewski M (2020). Inhibition of Bruton tyrosine kinase in patients with severe COVID-19. Sci. Immunol..

[80] Honda F (2012). The kinase Btk negatively regulates the production of reactive oxygen species and stimulation-induced apoptosis in human neutrophils. Nat. Immunol..

[81] Davis MJ, Lionakis MS (2018). B cells protect T cell-deficient mice from cryptococcal brain invasion. Virulence.

[82] Dufaud C, Rivera J, Rohatgi S, Pirofski LA (2018). Naive B cells reduce fungal dissemination in *Cryptococcus neoformans* infected Rag1(-/-) mice. Virulence.

[83] Taramasso L, Tatarelli P, Di Biagio A (2016). Bloodstream infections in HIV-infected patients. Virulence.

[84] Zaragoza O (2010). Fungal cell gigantism during mammalian infection. PLoS Pathog..

[85] da Silva-Junior EB (2021). The role of Toll-like receptor 9 in a murine model of *Cryptococcus gattii* infection. Sci. Rep..

[86] Mukaremera L (2018). Titan cell production in *Cryptococcus neoformans* reshapes the cell wall and capsule composition during infection. Cell. Surf..

[87] Davis MJ (2019). Pulmonary iron limitation induced by exogenous type I IFN protects mice from *Cryptococcus gattii* independently of T cells. MBio.

[88] Trevijano-Contador N (2018). *Cryptococcus neoformans* can form titan-like cells in vitro in response to multiple signals. PLoS Pathog..

[89] Garcia-Barbazan I (2016). The formation of titan cells in *Cryptococcus neoformans* depends on the mouse strain and correlates with induction of Th2-type responses. Cell Microbiol..

[90] Guess T (2018). Size matters: Measurement of capsule diameter in *Cryptococcus neoformans*. J. Vis. Exp..

